# Antibiotic Prophylaxis in Surgery: Current Insights and Future Directions for Surgical Site Infection Prevention

**DOI:** 10.7759/cureus.47858

**Published:** 2023-10-28

**Authors:** Simran Dhole, Chandrashekhar Mahakalkar, Shivani Kshirsagar, Abhilasha Bhargava

**Affiliations:** 1 General Surgery, Jawaharlal Nehru Medical College, Datta Meghe Institute of Higher Education and Research, Wardha, IND

**Keywords:** surgical medicine, antibiotic resistance, evidence-based practices, ssi prevention, antibiotic prophylaxis, surgical site infections

## Abstract

Surgical site infections (SSIs) remain a significant concern in the field of surgery, contributing to patient morbidity, prolonged hospital stays, and increased healthcare costs. Antibiotic prophylaxis, the administration of antibiotics before surgery, has been a cornerstone in preventing SSIs for decades. This review explores the current state of antibiotic prophylaxis in surgery, offering insights into its effectiveness, challenges, and emerging trends. In this comprehensive analysis, we delve into the historical development of antibiotic prophylaxis, examining its evolution from early practices to modern guidelines. We explore the various classes of antibiotics commonly used, their dosing regimens, and the importance of timing in optimizing prophylactic interventions. Additionally, we investigate the role of patient-specific factors, such as comorbidities and allergies, in tailoring antibiotic prophylaxis to individual needs. While antibiotic prophylaxis has undeniably reduced the incidence of SSIs, concerns about antimicrobial resistance and adverse effects necessitate a reevaluation of current practices. This review presents a critical assessment of the challenges posed by the overuse and misuse of antibiotics in surgery and highlights the urgent need for judicious antibiotic stewardship. Moreover, the future of antibiotic prophylaxis holds promise with the emergence of innovative strategies such as antimicrobial coatings, probiotics, and immunomodulatory agents. We discuss these novel approaches and their potential to enhance SSI prevention while minimizing antibiotic-related risks. In conclusion, antibiotic prophylaxis in surgery has been instrumental in reducing SSIs, but its continued effectiveness requires a multifaceted approach. By addressing current challenges, promoting antibiotic stewardship, and embracing innovative strategies, we can advance the field of SSI prevention and improve patient outcomes in the years to come. This review provides valuable insights and direction for clinicians, researchers, and policymakers as they navigate the evolving landscape of surgical prophylaxis.

## Introduction and background

Surgical site infections (SSIs) represent a significant and preventable complication in the field of surgery, posing substantial risks to patients and placing a substantial economic burden on healthcare systems. An SSI is a nosocomial infection at or near a surgical incision or operative site within 30 days following surgery or within 90 days if prosthetic material is implanted during surgery. SSIs can manifest as superficial infections involving the skin and subcutaneous tissue, deep infections affecting fascial and muscle layers, or organ/space infections involving any body part other than the incision site. These infections are characterized by localized signs and symptoms of inflammation, such as redness, swelling, warmth, and purulent discharge, and are often associated with systemic manifestations, including fever and leukocytosis [1-4].

Firstly, SSIs can lead to substantial patient morbidity, prolong hospital stays, and, in severe cases, result in mortality. Moreover, SSIs may require additional surgical procedures, such as debridement or implant removal, to effectively manage the infection. Secondly, these infections place a considerable financial burden on healthcare systems due to increased hospitalization costs, prolonged antibiotic therapy, and potential legal liabilities. Consequently, mitigating SSIs improves patient outcomes and contributes to cost-effective healthcare delivery [5,6]. Antibiotic prophylaxis, administering antibiotics before surgery, is pivotal in preventing SSIs. The rationale behind this practice is to reduce the microbial load at the surgical site, thereby minimizing the risk of infection. Properly selected antibiotics, administered at the appropriate time and in the correct dosage, have significantly decreased the incidence of SSIs. However, the use of antibiotics for prophylaxis is challenging, including concerns about antibiotic resistance and potential adverse effects. Therefore, understanding the nuances of antibiotic prophylaxis is critical to optimizing its benefits while minimizing risks [7].

## Review

Surgical site infections (SSIs)

Types and Classifications of SSIs

Superficial SSIs: These infections are primarily localized to the outermost layers of the surgical site, namely the skin and subcutaneous tissue. They often manifest with visible signs of infection, such as redness, swelling, warmth, and purulent discharge. While superficial SSIs are generally less invasive than their deeper counterparts, they should be considered. If left untreated, they can progress, leading to more severe complications or even the development of deep or organ/space SSIs [8].

Deep SSIs: In contrast to superficial SSIs, deep SSIs extend beyond the superficial layers of the surgical site, reaching the fascial and muscle layers beneath the incision. These infections are characterized by their invasiveness, potentially causing complications such as the formation of abscesses, tissue necrosis, or damage to vital structures. Deep SSIs are associated with a higher degree of morbidity, often requiring prolonged hospitalization, additional surgical procedures, and intensive medical management to address the deep-seated infection and prevent further harm to the patient [9].

Organ/space SSIs: Organ/space SSIs encompass infections affecting any body part other than the incision site. These infections can be particularly challenging to diagnose and manage due to their potential involvement of vital organs or body cavities. Organ/space SSIs may require surgical intervention, such as drainage procedures or reoperation, and extended courses of antibiotic therapy to clear the infection effectively. Given their complexity and potential for severe consequences, these infections demand close monitoring and a multidisciplinary approach to treatment [10].

Epidemiology and Prevalence of SSIs

Incidence rates: SSIs are common healthcare-associated infections in surgical patients, with varying rates depending on factors such as the type of surgery, patient health, and adherence to infection prevention protocols. Invasive procedures and prosthetic implants pose higher SSI risks [11].

Risk factors: Patient-specific and procedure-related factors increase SSI susceptibility. Immunosuppression from medical conditions or medications weakens the body's defenses. Conditions such as obesity and diabetes hinder wound healing and promote bacterial growth. Longer surgeries increase infection risk, especially for contaminated or dirty wounds. Foreign bodies such as surgical implants can also increase the risk, necessitating preventive measures [12].

Economic impact: SSIs not only harm patient health but also strain healthcare systems economically. Treatment costs go beyond the hospital stay and include additional surgeries, diagnostic tests, and prolonged antimicrobial therapy. Legal battles can further escalate healthcare costs [13].

Global variation: SSIs' prevalence varies globally due to healthcare resources, sanitation practices, and facility quality. Developing countries with limited resources and poor sanitation may have higher SSI rates. Overcrowded healthcare settings complicate understanding and managing SSIs in these regions [1].

Impact on Patient Outcomes and Healthcare Costs

Patient morbidity: SSIs cause significant suffering and reduced quality of life for patients. They affect emotional and psychological well-being, leading to extended healing processes, multiple healthcare facility visits, and continued reliance on healthcare resources [14].

Prolonged hospitalization: SSIs result in longer hospital stays, which increase patient discomfort and strain healthcare resources. This strain can lead to delays in elective surgeries and affect the entire healthcare system [15].

Mortality: While not always the direct cause of death, SSIs can contribute to mortality, especially in vulnerable populations. Complications from SSIs weaken patients and make them less able to handle other health challenges, making prevention crucial, especially in high-risk groups [16].

Healthcare costs: SSIs impose a significant economic burden on healthcare systems. Direct costs include infection treatment expenses, additional surgeries, extended hospital stays, tests, and antimicrobial therapy. Indirect costs involve lost productivity due to extended hospitalization and potential legal issues in severe cases. Effective prevention strategies such as antibiotic prophylaxis are essential to redirect resources to other areas of patient care, research, and development [17].

Historical perspective

Evolution of Antibiotic Prophylaxis in Surgery

Pre-antibiotic era: The pre-antibiotic era was characterized by surgical procedures fraught with a high risk of infection and dire patient consequences. Surgeons face formidable challenges in combating postoperative infections, primarily due to ineffective antimicrobial agents. Without antibiotics, the surgical arena was a battlefield against invisible adversaries, where even routine procedures carried a substantial risk of infection. Patients frequently succumbed to severe complications, and high morbidity and mortality rates marred the outcomes of surgeries. The lack of tools to control bacterial proliferation in surgical wounds cast a shadow over the field of surgery, highlighting the pressing need for a breakthrough [18].

Discovery of antibiotics: The mid-20th century witnessed the groundbreaking discovery of antibiotics, a medical revolution that transformed surgical practice. The advent of penicillin and subsequent antibiotics provided hope for surgeons grappling with the scourge of SSIs. Antibiotics emerged as powerful weapons in the arsenal against infectious complications, ushering in a new era of proactive infection control. For the first time, surgeons had tools to combat bacteria directly, significantly reducing postoperative complications. This newfound ability to administer antibiotics prophylactically before surgical incisions marked a profound turning point, ultimately enhancing patient safety and reshaping the surgery landscape [19].

Early Successes and Challenges

Initial successes: The initial application of antibiotics as prophylactic agents in surgical procedures yielded striking successes. These early triumphs were evident in the substantial reduction of SSIs, which had long plagued surgical practice. Surgeons and patients alike marveled at the tangible benefits as SSIs became less frequent and their associated morbidity and mortality rates plummeted. The introduction of antibiotics instilled newfound hope, transforming surgical interventions into safer and more reliable endeavors [20].

Overuse and resistance: Despite the remarkable achievements, the euphoria surrounding antibiotics precipitated a darker issue. The widespread and indiscriminate administration of antibiotics, sometimes in situations where they confer minimal benefit, sowed the seeds of a formidable adversary: antibiotic resistance. The emergence of antibiotic-resistant strains of bacteria represents an enduring challenge in modern healthcare. The overreliance on these powerful drugs inadvertently fostered the survival of resistant organisms, diminishing the effectiveness of antibiotics over time. This challenge reverberates across various medical contexts, including surgical prophylaxis, reminding us of the importance of judicious antibiotic use [21].

Challenges in selection: The selection of appropriate antibiotics for prophylaxis introduced yet another layer of complexity. Surgeons faced the daunting task of choosing antibiotics with an optimal spectrum of activity capable of targeting likely pathogens encountered during specific surgical procedures. This decision-making process necessitated a delicate balance between efficacy and minimizing the risk of adverse effects. Factors such as patient allergies, potential drug interactions, and evolving bacterial resistance patterns further compounded the challenge of antibiotic selection, underscoring the need for a nuanced and evidence-based approach [22].

Development of Guidelines and Protocols

Emergence of guidelines: As antibiotic prophylaxis became evident, healthcare organizations and professional societies stepped forward to provide much-needed guidance. These organizations, including the American College of Surgeons (ACS), the Centers for Disease Control and Prevention (CDC), and the World Health Organization (WHO), began issuing comprehensive guidelines. These guidelines were meticulously crafted, drawing upon a wealth of clinical evidence to offer evidence-based recommendations on various aspects of antibiotic prophylaxis [23].

Protocol implementation: The adoption of guidelines by hospitals and surgical teams marked a significant shift in clinical practice. Protocols based on these guidelines became the standard of care for surgical procedures. They stipulated the precise timing of antibiotic administration, emphasizing the importance of giving antibiotics shortly before incision to ensure that therapeutic levels were present when the patient was most vulnerable to infection. Additionally, protocols underscored the discontinuation of antibiotics within 24 hours of surgery, a crucial step in minimizing the risk of antibiotic resistance [24].

Quality improvement initiatives: Recognizing the need for ongoing monitoring and improvement, healthcare institutions established quality improvement programs. These initiatives were designed to systematically track guideline adherence and assess their real-world impact on SSI rates. By collecting and analyzing data, institutions could identify areas of improvement, implement corrective measures, and ensure that best practices are consistently applied throughout their surgical departments [25].

Ongoing updates: The dynamic nature of medicine, including antimicrobial pharmacology and bacterial resistance patterns, necessitated regularly updating guidelines. To remain practical, guidelines needed to evolve in response to emerging scientific evidence and the ever-changing landscape of surgical practice. Regular updates ensure that practitioners stay aligned with the latest knowledge, allowing them to adapt their prophylactic approaches to new challenges and opportunities [26].

Current practices

Guidelines for Antibiotic Prophylaxis in Surgery

Professional societies: Leading professional organizations, including ACS, CDC, and WHO, are instrumental in providing comprehensive guidelines for antibiotic prophylaxis in surgical settings. These organizations leverage their expertise to synthesize the latest scientific evidence into practical recommendations that healthcare professionals can readily implement [27].

Procedure-specific recommendations: Recognizing that not all surgical procedures carry the same risk of SSIs, these guidelines tailor their recommendations to specific types of surgeries. They consider the nuances of each procedure, such as the surgical site, patient characteristics, and surgical techniques employed. This individualized approach ensures that prophylactic antibiotic choices align closely with the unique infection risks of different surgeries [28].

Selection of antibiotics: The guidelines are meticulous in selecting appropriate antibiotics for prophylaxis. They prioritize antibiotics with the optimal spectrum of activity against the likely pathogens encountered during specific surgical procedures. Furthermore, these guidelines consider local antibiotic resistance patterns, ensuring that the antibiotics chosen remain effective in the face of regional resistance trends. This strategic antibiotic selection enhances the prophylactic effect and contributes to antibiotic stewardship by minimizing unnecessary broad-spectrum use [29].

Timing and Administration of Antibiotics

Timing: Administering antibiotics at the right time is fundamental. Antibiotics should be given within a specific timeframe before surgical incision, ideally 30-60 minutes before the procedure commences. This timing is strategically chosen to ensure that therapeutic levels of the antibiotic are present in the patient's tissues precisely when potential bacterial exposure occurs, typically when the surgical incision is made [30].

The rationale behind this precise timing is twofold. First, it allows antibiotics to reach the surgical site and adjacent tissues in concentrations that effectively combat any bacteria introduced during the procedure. Second, it aligns with the principle that antibiotic prophylaxis is most effective when the antibiotic's mechanism of action targets actively dividing bacteria, which are more vulnerable to antimicrobial agents. Administering antibiotics shortly before incision significantly increases the likelihood of eradicating these bacteria [31].

Intraoperative dosing: The timing and duration of antibiotic administration during surgical procedures play a crucial role in infection prevention. While the initial preoperative dose is vital, it is important to consider that many surgical procedures may extend beyond the antibiotic's effective coverage window. In such cases, intraoperative dosing becomes a recommended practice, especially for surgeries lasting longer than 2-4 hours. Intraoperative dosing entails the administration of an additional dose of prophylactic antibiotics during the surgery, and it is employed to address the potential decline in antibiotic concentration over time. This supplementary dose is strategically administered to ensure that therapeutic levels of the antibiotic are maintained in the patient's tissues throughout the entire surgical procedure. Doing so guarantees continued protection against potential bacterial contamination and reduces the risk of SSIs [32]. Moreover, in some cases, local administration of antibiotics intraoperatively is adopted. This approach involves directly applying antibiotics to the specific site of the surgical incision or area of potential infection. The local administration of antibiotics during surgery serves as an additional layer of defense, providing enhanced protection at the immediate source of concern. This practice is particularly valuable when there is a higher risk of infection due to the nature of the surgery or the patient's unique circumstances. It complements systemic antibiotic administration and contributes to comprehensive infection control strategies in the operating room [33].

Selection of Appropriate Antibiotics

First-line agents: At the heart of antibiotic selection lies the preference for first-line agents, such as cefazolin. These antibiotics are favored for many surgical procedures due to their attributes, including a broad spectrum of activity against common pathogens encountered in surgery and a well-established safety profile. First-line antibiotics balance effectiveness and minimize the risk of adverse effects, making them the cornerstone of prophylaxis in various surgical settings. Their use reflects a conscious effort to provide patients with optimal protection against SSIs while ensuring safety [33].

Allergies and sensitivities: The complexity of antibiotic prophylaxis deepens when patients present with allergies or sensitivities to certain antibiotics. In such cases, a critical aspect of antibiotic selection is to assess and document the patient's history of allergies meticulously. Identifying known allergies is imperative to avoid adverse reactions. When patient allergies are identified, healthcare providers must pivot to alternative antibiotics with a similar activity spectrum against likely pathogens. These alternative agents must be chosen judiciously, considering the patient's allergies and sensitivities. The goal remains unaltered: to protect against SSIs without compromising patient safety [34].

Complex cases: Some surgical scenarios deviate from standard procedures, necessitating customized antibiotic regimens. These unique cases include surgeries involving the implantation of prosthetic materials or gastrointestinal procedures. The reason behind considering gastrointestinal surgery challenging in terms of SSI prophylaxis is primarily due to the distinct infection risks associated with these procedures. In such cases, guidelines offer precise recommendations for selecting and administering antibiotics to address this complexity. By providing tailored strategies for complex surgical procedures, these guidelines ensure that prophylactic antibiotics are aligned with the specific requirements of each surgery, thereby maximizing their effectiveness in preventing SSIs [35].

Duration of Prophylactic Treatment

In the majority of surgical procedures, a single prophylactic antibiotic dose administered shortly before the surgical incision is typically sufficient. This single-dose approach aligns with the principle of minimizing antibiotic exposure while ensuring that adequate antibiotic levels are present at the surgical site during the crucial initial stages of the procedure. This strategy aims to decrease the risk of selecting antibiotic-resistant bacterial strains in the patient's microbiome [36]. Prophylactic antibiotic use has gained widespread endorsement in surgical guidelines, effectively reducing the rates of SSI by restricting the administration of prophylactic antibiotics to a single dose. Adhering to this guideline enables healthcare providers to contribute to the battle against antibiotic resistance while lowering the potential for antibiotic-related adverse events and complications [37]. Surgical procedures involving the heart, such as coronary artery bypass grafting or heart valve surgery, often require extended prophylactic antibiotic treatment. The heightened susceptibility of cardiac tissues to infections and the prolonged duration of these surgeries necessitate a more extended course of antibiotics to ensure adequate protection for the patient [38]. In surgical cases involving the gastrointestinal tract, particularly those with ongoing contamination (e.g., perforated bowel or bowel obstruction), more extended periods of prophylaxis are frequently recommended. The risk of infection is elevated due to the introduction of gut bacteria into the surgical site. As a result, antibiotics may be administered for an extended period, sometimes up to 24 hours, to provide continuous protection [39].

Adherence to Guidelines in Clinical Practice

Multidisciplinary approach: The successful implementation of antibiotic prophylaxis guidelines necessitates a coordinated, multidisciplinary approach within the healthcare team. Collaboration among surgeons, anesthesiologists, nurses, and pharmacists is essential to optimize the administration of antibiotics. Surgeons and anesthesiologists must align surgical schedules with the timing of antibiotic administration, ensuring that antibiotics are given within the recommended timeframe before incision. Nurses are crucial in administering antibiotics and monitoring patients for adverse reactions or complications. Pharmacists contribute by ensuring the appropriate selection, dosing, and preparation of antibiotics and conducting medication reconciliation to avoid potential drug interactions. This collective effort ensures that antibiotic prophylaxis is seamlessly integrated into the surgical process, minimizing SSI risk [40].

Monitoring and quality improvement: Healthcare institutions recognize the significance of guideline adherence and often establish robust monitoring and quality improvement programs. These initiatives serve as a vital feedback loop, allowing healthcare systems to assess compliance with antibiotic prophylaxis guidelines and track SSI rates. Continuous monitoring helps identify areas where adherence may be lacking or improvements can be made. By scrutinizing these data, healthcare institutions can implement targeted interventions and interventions to address compliance issues. This proactive approach contributes to refining practices, ensuring that adherence to guidelines becomes an ingrained part of the institutional culture. It also provides valuable insights into the impact of adherence on SSI rates, allowing for data-driven improvements in patient care [41].

Education and training: Ongoing education and training programs for healthcare professionals are fundamental to successful adherence to antibiotic prophylaxis guidelines. These programs aim to raise awareness of the importance of guidelines and promote best practices among surgical teams. Healthcare professionals need regular updates on the latest guidelines, as recommendations may evolve based on new evidence and emerging trends in antibiotic resistance. Education also addresses common misconceptions and myths about antibiotic prophylaxis, ensuring that healthcare providers understand the guidelines' rationale. Training programs extend beyond just understanding guidelines; they also focus on practical aspects such as proper antibiotic administration techniques and recognizing potential adverse reactions. By investing in the education and training of healthcare professionals, institutions empower their teams to make informed decisions and deliver the highest standard of care, ultimately contributing to more effective SSI prevention through guideline adherence [42].

Mechanisms of action

How Antibiotics Prevent SSIs

A key consideration in selecting antibiotics for surgical procedures is their pharmacokinetics, especially their distribution to the incision tissue. Antibiotics are chosen meticulously, taking into account their specific affinity for targeting particular groups of bacteria commonly encountered in surgical settings. These selected antibiotics are pivotal in disrupting critical bacterial processes, such as cell wall synthesis, protein production, DNA replication, and other vital functions. This deliberate targeting ensures that the antibiotics effectively eliminate or inhibit the bacteria responsible for SSIs while preserving the beneficial microorganisms [43]. By considering the pharmacokinetics, we ensure that the antibiotics are delivered optimally to the incision site, thereby enhancing their effectiveness in preventing SSIs [44].

Reducing bacterial load: A fundamental aspect of antibiotic prophylaxis involves substantially reducing bacterial populations at the surgical site. By decreasing the number of bacteria in the vicinity, antibiotics create a less favorable environment for establishing infections. This is particularly critical during the initial stages of wound healing when the surgical site is most vulnerable to bacterial invasion [45].

Minimizing local spread: Antibiotics act as a formidable barrier against the local spread of bacteria beyond the confines of the surgical incision. They effectively curtail the migration of bacteria to deeper tissue layers or body cavities, where infections can become more severe and challenging to manage. Antibiotics help prevent the development of deep tissue and organ/space infections by containing bacteria at the incision site [37].

Immune system support: Antibiotics indirectly support the patient's immune system in its battle against potential infections. Antibiotics afford the immune system a favorable environment by reducing the bacterial burden within the surgical area. This immune system support can enhance the body's natural defense mechanisms, allowing it to focus on clearing any remaining bacteria and expediting the healing process [46].

Factors Influencing Antibiotic Efficacy

Timing: The timing of antibiotic administration is of paramount importance. Antibiotics should be administered shortly before surgical incision, ideally within 30 to 60 minutes. This precise timing ensures that therapeutic antibiotic levels are present at the surgical site when the patient is most vulnerable to potential bacterial exposure. Early administration is crucial because delayed dosing can reduce antibiotic efficacy [30].

Dosage and route of administration: Accurately administering antibiotics is crucial for optimal prophylaxis. It involves not only the correct dosage but also the appropriate route of administration. To ensure adequate tissue concentrations capable of inhibiting bacterial growth, the dosage should be meticulously calculated, considering factors such as body weight, renal function, and the volume of distribution. These factors collectively influence the selection of the most suitable route of administration, whether oral, intravenous, intramuscular, or another method. Under-dosing can render antibiotics ineffective, while excessive dosing may increase the risk of adverse effects without necessarily enhancing prophylactic benefits [47].

Spectrum of activity: Antibiotics should possess a spectrum of activity broad enough to cover the likely pathogens associated with the specific surgical procedure. Tailoring antibiotic choice to the anticipated pathogens is crucial for efficacy. However, the selection of overly broad-spectrum antibiotics can be counterproductive, potentially contributing to the development of antibiotic resistance. Thus, a careful balance must be struck between targeted coverage and minimizing the risk of resistance [48].

Patient factors: Patient-related variables, such as allergies, comorbidities, and immunosuppression, can significantly influence antibiotic prophylaxis efficacy. Knowledge of a patient's medical history and potential allergens is essential to selecting antibiotics that are both effective and safe. Patients with underlying health conditions may require antibiotic choice or dosage adjustments to ensure adequate prophylaxis [37].

Surgical technique: The surgical technique can substantially impact antibiotic efficacy. Adequate tissue perfusion, proper wound closure, and meticulous aseptic practices are essential to maximizing antibiotic prophylaxis's effectiveness. Ensuring that the surgical environment is as free from contamination as possible and that wounds are well-approximated help prevent bacterial entry and subsequent infection [49].

Role of Antibiotic Resistance

Selection pressure: The ubiquitous use of antibiotics, including their prophylactic administration, exerts relentless pressure on bacteria. This pressure encourages the survival and proliferation of bacterial strains that can withstand antibiotic exposure. Over time, these survivors develop genetic adaptations that confer resistance, rendering antibiotics less effective in subsequent encounters [50].

Multidrug resistance: The most alarming manifestation of antibiotic resistance is the emergence of multidrug-resistant organisms (MDROs). These tenacious pathogens have evolved mechanisms to resist multiple classes of antibiotics, severely limiting treatment options. In surgical settings, encountering MDROs can lead to dire clinical challenges, heightening the risk of untreatable infections [51].

Infection risk: Patients who harbor or are infected with antibiotic-resistant bacteria have an elevated risk of SSIs. Once stalwart defenders against infection, prophylactic antibiotics may prove less efficacious in the face of these resilient pathogens. Consequently, SSIs caused by antibiotic-resistant bacteria can be more challenging to manage and may lead to poorer patient outcomes [52].

Balancing act: The crux lies in achieving a delicate balance. On the one hand, effective antibiotic prophylaxis remains pivotal in preventing SSIs and their associated complications. On the other hand, the overuse or misuse of antibiotics amplifies the peril of antibiotic resistance. Antibiotic stewardship programs, a linchpin of responsible antibiotic use, seek to harmonize these conflicting imperatives. By optimizing antibiotic use, these programs strive to curtail resistance while ensuring that antibiotic prophylaxis remains a potent safeguard against SSIs [53]. Table 1 enlists the examples of antibiotics and their mechanisms of action [21,27,33,40,48].

**Table 1:** Examples of antibiotics and their mechanisms of action. [21,27,33,40,48]

Antibiotic Name	Antibiotic Type	Mechanism of Action
Amoxicillin	Penicillin	Inhibits bacterial cell wall synthesis by targeting peptidoglycan.
Ciprofloxacin	Fluoroquinolone	Inhibits DNA gyrase, preventing DNA replication and transcription.
Doxycycline	Tetracycline	Interferes with protein synthesis by binding to the 30S ribosomal subunit.
Vancomycin	Glycopeptide	Inhibits cell wall synthesis in Gram-positive bacteria by binding to the peptidoglycan precursor.
Azithromycin	Macrolide	Blocks protein synthesis by binding to the 50S ribosomal subunit.
Trimethoprim-sulfamethoxazole	Sulfonamide	Inhibits folic acid synthesis in bacteria by targeting dihydrofolate reductase.
Gentamicin	Aminoglycoside	Disrupts protein synthesis by binding to the 30S ribosomal subunit.
Linezolid	Oxazolidinone	Inhibits bacterial protein synthesis by binding to the 50S ribosomal subunit.

Table 2 enumerates the organizations that address antibiotic resistance with its clinical relevance [27,50,51].

**Table 2:** Organizations addressing antibiotic resistance [27,50,51] AMR, antimicrobial resistance

Organization	Significance	Relevance	Impact
World Health Organization	Leading international health organization with a critical role in global health security and AMR management.	Develops guidelines for prudent antibiotic use, monitors resistance patterns, and supports countries in AMR action plans.	WHO's Global Antimicrobial Resistance Surveillance System (GLASS) aids countries in making evidence-based decisions regarding antibiotics.
Centers for Disease Control and Prevention	Key player in the U.S. efforts to combat antibiotic resistance.	Works on surveillance, prevention, and education to reduce resistance.	CDC's Antibiotic Resistance Solutions Initiative helped reduce antibiotic-resistant infections in healthcare settings by 18%.
The Wellcome Trust	Global charitable foundation funding research in various fields, including AMR.	Supports research on new antibiotics, alternative treatments, and diagnostic tools.	Wellcome's investments have accelerated the development of novel antibiotics, such as teixobactin, showing promise against resistant bacteria.
Pew Charitable Trusts	Known for advocacy efforts to combat antibiotic resistance.	Works on policy advocacy and public awareness campaigns to promote responsible antibiotic use.	Pew's Antibiotic Resistance Project contributed to the passing of the 2016 U.S. National Action Plan for Combating Antibiotic-Resistant Bacteria.

Efficacy and effectiveness

Clinical Studies and Evidence Supporting Antibiotic Prophylaxis

Randomized controlled trials (RCTs): Across a broad spectrum of surgical procedures, numerous RCTs have yielded consistent and compelling results. These studies demonstrate that antibiotic prophylaxis significantly reduces the incidence of SSIs. RCTs provide a robust foundation for the efficacy of prophylactic antibiotics, offering scientific rigor and minimizing biases that can influence outcomes. The rigorous design of RCTs ensures that the observed benefits can be confidently attributed to antibiotic prophylaxis [54].

Procedure-specific evidence: Clinical trials often drill down to the nuances of specific surgical procedures, tailoring their investigations to colorectal surgery, orthopedic joint replacements, cesarean sections, and more. Procedure-specific evidence enhances our understanding of the effectiveness of antibiotic prophylaxis within distinct clinical contexts. This approach acknowledges that different surgeries may present with varying infection risks, patient populations, and bacterial flora, necessitating tailored prophylactic strategies. As a result, surgeons can make informed decisions about antibiotic selection and dosages to maximize the protective benefits for their patients [55].

Meta-analyses: Beyond individual trials, meta-analyses offer a comprehensive synthesis of data from multiple RCTs. By pooling data across studies, meta-analyses provide a powerful overview of the overall benefit of antibiotic prophylaxis. These analyses help distill patterns and trends, lending further weight to the efficacy of prophylactic antibiotics. Importantly, meta-analyses accommodate the diversity of patient populations and surgical settings, offering a global perspective on the preventive impact of antibiotic prophylaxis [56].

National and international guidelines: The recommendations from influential healthcare organizations, such CDC and WHO, are intricately informed by a thorough review of clinical evidence. These guidelines encapsulate a consensus view, aligning with the findings of clinical studies and RCTs. They serve as the cornerstone for best practices in antibiotic prophylaxis. The fact that these authoritative bodies unequivocally advocate for antibiotic prophylaxis underscores its critical role in safeguarding surgical patients from SSIs [57].

Real-world observations: While RCTs and clinical trials provide controlled environments for evaluating interventions, real-world practice settings offer invaluable insights into the practical impact of antibiotic prophylaxis. Observational studies conducted in clinical practice settings have consistently corroborated the effectiveness of antibiotic prophylaxis. They demonstrate reductions in SSI rates when surgical teams adhere to established guidelines. These real-world observations underscore the translatable benefits of antibiotic prophylaxis, reflecting its pragmatic utility in diverse healthcare settings [58].

Reduction in morbidity and mortality: Perhaps the most compelling evidence of antibiotic prophylaxis' efficacy emanates from its palpable impact on patient outcomes. Antibiotic prophylaxis significantly reduces patient morbidity, mortality, and healthcare costs linked to SSIs when appropriately administered. Beyond statistical significance, these tangible improvements in patient well-being are a poignant reminder of the profound value of antibiotic prophylaxis in surgical care. It reaffirms its status as an indispensable tool in the relentless pursuit of patient safety and surgical excellence [59].

Risks and Side Effects of Antibiotic Prophylaxis

Allergic reactions to antibiotics can range in severity, from mild skin rashes to life-threatening anaphylactic shock. The identification of patient allergies and the acquisition of a comprehensive medical history are crucial in managing this risk. In cases of known allergies, choosing alternative antibiotics with different chemical structures can help ensure patient safety while still effectively preventing SSIs [34]. Antibiotics, despite their benefits, can unintentionally disrupt the balance of beneficial bacteria in the gastrointestinal tract. This disruption can result in gastrointestinal disturbances such as diarrhea, abdominal pain, and nausea. These symptoms can cause discomfort for the patient, extend hospital stays, and impede postoperative recovery [60].

Prolonged or extensive use of antibiotics, even for prophylaxis, significantly increases the risk of developing *Clostridium difficile* infection (CDI). This potentially severe and recurrent gastrointestinal infection is caused by the overgrowth of C. difficile bacteria, often due to the disruption of the gut microbiome by antibiotics. CDI can lead to severe diarrhea, colitis, and even life-threatening complications [61]. An overarching concern associated with antibiotic prophylaxis is the risk of antibiotic resistance. The overuse and inappropriate use of antibiotics, including their prophylactic use, contribute to the emergence of antibiotic-resistant bacterial strains. Such resistance renders previously effective antibiotics ineffective, jeopardizing the treatment of SSIs and other infections. This emphasizes the need for a prudent approach to antibiotic use [62].

Certain antibiotics may interact with other medications that patients are taking concurrently. These interactions can alter the pharmacokinetics of both the antibiotic and the other drugs, potentially affecting their effectiveness or safety. Healthcare providers must remain vigilant in assessing potential drug interactions to prevent adverse outcomes [63]. The indiscriminate use of broad-spectrum antibiotics in prophylaxis can inadvertently promote the selection of antibiotic-resistant organisms. These MDROs pose a significant public health threat, as they limit treatment options and complicate the management of SSIs and other infections. Therefore, efforts to minimize the use of overly broad-spectrum antibiotics in prophylaxis are essential in addressing the emergence of MDROs [64].

Areas of Improvement in Current Practices

Antibiotic stewardship: Implementing robust antibiotic stewardship programs within healthcare institutions is imperative. These programs aim to ensure the judicious and appropriate use of antibiotics for SSI prevention across all healthcare settings. By carefully monitoring and regulating antibiotic use, healthcare providers can reduce the risk of antibiotic resistance. Such programs involve continuous evaluation of prescribing practices, monitoring of resistance patterns, and the development of guidelines that emphasize the responsible use of antibiotics. They promote a delicate balance between preventing SSIs and safeguarding the long-term effectiveness of antibiotics [65].

Tailored approaches: Recognizing that each patient is unique, conducting further research into patient-specific factors that can influence the efficacy and safety of antibiotic prophylaxis is essential. Age, comorbidities, immune status, and individual pharmacokinetics all play crucial roles in determining the optimal antibiotic regimen for a given patient. Tailoring antibiotic choices and dosages based on these factors can maximize the benefits of prophylaxis while minimizing the risks. This personalized medicine approach enhances SSI prevention and reduces potential adverse events and antibiotic resistance [66].

Alternative strategies: Exploring and implementing alternative strategies for SSI prevention represents a promising avenue for reducing reliance on antibiotics. Antimicrobial-coated implants, for example, can help minimize the risk of infections associated with surgical devices. Immunomodulatory agents that enhance the patient's immune response may complement antibiotic prophylaxis, particularly in cases where infections are challenging to prevent solely with antibiotics. Additionally, enhanced perioperative infection control measures, such as strict adherence to aseptic techniques and advanced wound care practices, can reduce the risk of SSIs without additional antibiotics [67].

Surveillance and feedback: Monitoring SSI rates and guideline adherence is essential for quality improvement in surgical practices. Healthcare institutions should establish comprehensive surveillance systems to track SSI occurrences and identify potential areas for improvement. Regular feedback loops with surgical teams can help address lapses in adherence to best practices. By analyzing data and sharing outcomes, institutions can drive change, reduce variation in practice, and ultimately enhance SSI prevention efforts. This approach fosters a culture of accountability and continuous improvement within the healthcare system [68].

Education and training: Ensuring that healthcare professionals receive ongoing education and training on antibiotic prophylaxis guidelines and principles is paramount. Effective implementation of prophylactic measures relies on the knowledge and commitment of the healthcare workforce. Regular training programs can keep healthcare providers up-to-date with the latest guidelines, advances in pharmacology, and best practices for antibiotic prophylaxis. Furthermore, education can help raise awareness about the importance of adherence to guidelines and the responsible use of antibiotics, promoting a culture of patient safety and effective SSI prevention within healthcare institutions [69].

Challenges and controversies

Overuse and Misuse of Antibiotics

In the context of low-risk surgical procedures, it is essential to consider the appropriateness of antibiotic prophylaxis and assess the risk of SSIs. Determining when to administer antibiotics in these cases is crucial, as the potential benefits must be weighed against the associated risks. Antibiotics are sometimes prescribed unnecessarily, depleting valuable medical resources and contributing to the emergence of antibiotic-resistant bacteria. Overusing antibiotics in low-risk procedures offers limited benefits and poses a substantial risk to public health [70]. Therefore, it is imperative to establish clear criteria and guidelines for evaluating the SSI risk of each procedure and making informed decisions regarding antibiotic prophylaxis.

Non-compliance with guidelines: Another challenge lies in the practice variability among surgeons. Despite well-established guidelines outlining when and how antibiotic prophylaxis should be administered, deviations from these guidelines persist. Surgeons may need more awareness, clinical judgment, or personal preferences to diverge from established protocols. This inconsistency in guideline adherence can result in inappropriate antibiotic use, increasing the likelihood of antibiotic resistance and undermining the efficacy of prophylactic measures [71].

Pressure for prophylactic use: A complex dimension of overuse is the pressure patients and their families exert for prophylactic antibiotic administration. Patients may seek antibiotics as reassurance, believing that antibiotics will guarantee protection against SSIs. This pressure can lead to the administration of antibiotics even in cases where guidelines advise against it. While patient satisfaction and peace of mind are valid concerns, succumbing to such pressures can perpetuate unnecessary antibiotic use, further fueling the problem of antibiotic resistance [33].

Development of Antibiotic Resistance

Selection for resistant bacteria: The routine use of antibiotics as prophylactic measures in surgery has profound consequences for bacterial populations. It introduces a selective pressure favoring the survival and proliferation of bacteria with pre-existing resistance or those that acquire resistance mutations. The antibiotics act as a Darwinian filter, allowing resistant bacteria to thrive while susceptible ones are eliminated. This phenomenon is particularly concerning because it contributes significantly to the growing global problem of antibiotic resistance. The more antibiotics are used, especially when unnecessary, the more chances bacteria have to evolve mechanisms to evade these drugs [72].

MDROs: The emergence of MDROs represents a critical juncture in the evolution of antibiotic resistance. MDROs are bacteria that exhibit resistance to multiple classes of antibiotics, including those considered as last-resort treatments. The widespread use of antibiotics, including prophylactic use, plays a pivotal role in developing MDROs. When repeatedly exposed to various antibiotics, bacteria can accumulate resistance mechanisms, rendering them impervious to conventional therapeutic options. This situation poses a dire challenge for clinicians, as it limits the arsenal of antibiotics available to combat infections caused by MDROs [73].

Allergic Reactions and Other Adverse Events

Allergic reactions: Allergic reactions to antibiotics present a multifaceted challenge in SSI prevention. These reactions can manifest along a spectrum, ranging from mild skin rashes and itching to severe and life-threatening anaphylactic shock. The importance of identifying patient allergies cannot be overstated, as administering an antibiotic to which a patient is allergic can lead to dire consequences. To mitigate the risk, meticulous patient history-taking and allergy documentation are essential before any surgical antibiotic prophylaxis [74,75].

*Clostridium difficile* infection: The gut microbiome disruption due to prolonged antibiotic use, even when administered for prophylaxis, introduces another dimension of concern. This disruption can significantly increase the risk of CDI, a bacterial colon infection. CDI can manifest with severe diarrhea, abdominal pain, and, in some cases, life-threatening complications [76]. The relationship between CDI and prophylactic antibiotics highlights the intricate trade-offs involved in surgical practice. While antibiotics are administered to prevent SSIs, they can inadvertently create conditions conducive to CDI development. Healthcare providers must exercise antibiotic selection and duration discretion to address this challenge [77].

Economic considerations: The cost of antibiotics selected for prophylaxis can vary significantly, especially when considering newer or broader-spectrum agents. While these antibiotics may offer advantages in terms of their spectrum of activity, they often come with a substantial price tag. Healthcare providers face the challenge of balancing these antibiotics' clinical benefits against their economic costs. Striking this equilibrium is a continuous endeavor, requiring a nuanced assessment of the specific surgical context and patient population [62].

Prophylactic antibiotics are primarily administered to prevent SSIs, and their efficacy can lead to substantial savings in healthcare expenses. Antibiotic prophylaxis can deliver significant economic benefits by avoiding the need for additional surgeries, prolonged hospital stays, and costly treatment of postoperative infections. However, this apparent economic gain must be weighed against the long-term costs of antibiotic resistance. The emergence of resistant bacterial strains can lead to challenging and costly treatment scenarios, negating the initial savings [78].

Healthcare institutions often grapple with concerns related to legal liability in the context of SSIs. The prospect of litigation from patient infections acquired during surgery can be daunting. Some institutions may overreact to prophylactic antibiotics as legal protection in response to these legal worries. This practice can inadvertently contribute to antibiotic overuse and, in turn, fuel the problem of antibiotic resistance [79].

In specific healthcare systems, reimbursement models can inadvertently incentivize the use of antibiotics for prophylaxis. Preventing complications, including SSIs, can lead to financial bonuses for healthcare providers. While the intention behind these incentives is to improve patient outcomes, there is a risk that they may encourage the indiscriminate use of prophylactic antibiotics. Striking a balance between incentivizing quality care and avoiding unnecessary antibiotic administration is an ongoing challenge within these reimbursement structures [77].

Controversy Surrounding Antibiotic Duration

The controversy surrounding antibiotic duration is a subject of extensive discussion within the medical field, and it stems from the delicate equilibrium that must be established between effectively treating infections and curtailing the development of antibiotic resistance. The choice between short-term and long-term antibiotic regimens is at the heart of this controversy. Short-term treatment courses are often preferred due to their potential to minimize the risk of antibiotic resistance. The rationale here is that limiting bacteria's exposure to antibiotics diminishes the likelihood of them developing resistance. Consequently, shorter courses are commonly employed for routine infections where a swift and decisive response is highly effective [30,36].

However, the debate takes a more intricate turn when addressing severe or recurring infections. In these scenarios, the focus shifts toward longer antibiotic durations. Extended treatment courses are designed to ensure the thorough eradication of all infectious agents, reducing the risk of recurrent infections and the emergence of resistant strains. Striking the right balance between these two approaches becomes the central challenge in this debate. To bring clarity to this ongoing discussion, numerous reputable health organizations, including CDC, WHO, and the Infectious Diseases Society of America, have issued comprehensive guidelines and recommendations regarding antibiotic duration. These guidelines serve as invaluable references for healthcare practitioners, offering critical insights into the recommended treatment durations based on the type and severity of the infection. They guide navigating the complex terrain of antibiotic therapy duration, assisting healthcare providers in making well-informed decisions in various clinical scenarios [36,37].

Future directions

Innovations in Antibiotic Prophylaxis

Nanotechnology offers the potential to revolutionize antibiotic delivery in surgical prophylaxis with its precision and versatility. Nanoparticles can be designed to encapsulate antibiotics and release them slowly at the surgical site. This localized drug delivery minimizes systemic exposure, reducing the risk of adverse effects while maintaining therapeutic levels where they are most needed. This innovation enhances the efficacy of antibiotics and contributes to patient safety by minimizing the impact on the body's microbiome [80]. Combining antibiotics with different mechanisms of action is a strategy gaining traction in the quest for more effective prophylaxis. Using a combination of antibiotics, each targeting a specific aspect of bacterial growth and replication, reduces the risk of resistance development. This approach broadens the spectrum of activity while maintaining the delicate balance of antibiotic stewardship. As a result, prophylactic regimens become more robust against a broader range of potential pathogens, further safeguarding against SSIs [81].

The era of personalized medicine extends its reach into antibiotic prophylaxis. Pharmacogenomics, which involves tailoring drug choices based on an individual's genetic profile, may soon become a standard practice in surgical settings. By analyzing a patient's genetic makeup, healthcare providers can identify genetic markers influencing how the body metabolizes and responds to antibiotics. This knowledge allows for selecting effective antibiotics against likely pathogens and optimized for the patient's unique physiology. Personalized prophylaxis ensures that patients receive the most appropriate antibiotic, minimizing the risk of adverse reactions and enhancing overall efficacy [82]. To combat the growing threat of antibiotic resistance, healthcare institutions are exploring antibiotic rotation strategies. This involves periodically changing the antibiotics used for prophylaxis to prevent the overexposure of bacterial populations to specific drugs. By rotating antibiotics, the selection pressure for resistance is reduced, making it harder for bacteria to develop resistance mechanisms. While implementing rotation protocols requires careful planning and coordination, it represents a proactive approach to preserving the effectiveness of antibiotics for future surgical patients [83].

Personalized Medicine and Tailored Approaches

Recent research endeavors have delved into the intricate web of host factors that influence an individual's susceptibility to SSIs. These factors encompass many patient-specific characteristics, including genetics, immunological profiles, and microbiome composition. Genetic variations can predispose some individuals to a heightened risk of infections, while others may possess innate immunity that offers better protection. By identifying these host factors, clinicians can tailor prophylactic regimens to match a patient's unique vulnerabilities and strengths [84]. The development of biomarkers for predicting a patient's risk of SSI represents a significant stride toward personalized prophylaxis. Biomarkers are biological indicators that provide valuable insights into an individual's susceptibility to infections. Researchers are actively exploring a wide range of biomarkers, including markers of inflammation, immune function, and microbial colonization. Healthcare providers can stratify patients into risk categories by analyzing these biomarkers before surgery, enabling tailored prophylactic strategies. As biomarker profiles indicate, patients at higher risk may receive more intensive antibiotic prophylaxis, while those at lower risk may benefit from reduced antibiotic exposure [85].

Alternatives to Antibiotics for SSI Prevention

Antimicrobial coatings: In surgical implants and medical devices, developing enhanced implant coatings with antimicrobial properties represents a promising avenue for reducing the risk of SSIs. These coatings are designed to release antimicrobial agents gradually, creating a protective barrier at the implant site. These coatings can significantly lower the likelihood of infections associated with implanted devices by inhibiting bacterial colonization and biofilm formation [86].

Immunomodulatory therapies: Researchers are exploring immunomodulatory therapies as potential alternatives or adjuncts to antibiotic prophylaxis. These therapies aim to bolster the patient's immune response, empowering the body to fend off potential pathogens better. By optimizing the immune system's capacity to recognize and combat invading microorganisms, these treatments may offer a proactive defense against SSIs. While still in the experimental phase, immunomodulatory agents hold promise in reducing the need for antibiotics in preventing SSIs, particularly in cases where patient-specific factors make them susceptible to infections [87].

Probiotics and prebiotics: The gut microbiome plays a pivotal role in overall health and immunity. Manipulating this microbial ecosystem with probiotics (beneficial bacteria) and prebiotics (substances that promote their growth) is gaining attention as a strategy to reduce the risk of SSIs, particularly in gastrointestinal surgeries. Probiotics can enhance the balance of beneficial bacteria in the gut, strengthening the body's defenses against potential pathogens. Prebiotics provide nourishment for these helpful microbes, fostering a healthier gut environment. By fortifying the gut microbiome, probiotics and prebiotics may indirectly bolster the patient's overall immune response and, in turn, reduce the susceptibility to SSIs [88].

Bacteriophages: Bacteriophages, viruses that infect and selectively kill bacteria, offer a targeted and precise approach to combat specific bacterial pathogens. They have gained attention as potential alternatives to antibiotics for SSI prevention. Bacteriophages can be tailored to target specific bacteria responsible for SSIs, leaving beneficial bacteria unharmed. By directly attacking the causative agents of infection, bacteriophages may provide an effective means of prevention while minimizing the disruption of the broader microbiome. Research into the use of bacteriophages in surgery holds promise for a future where antibiotic prophylaxis is complemented or even replaced by this particular and adaptable approach [89].

Multidisciplinary Strategies for SSI Prevention

Multidisciplinary teams are pivotal in enhancing infection control measures within healthcare settings. Collaboration among surgeons, nurses, infection control specialists, and environmental services staff allows for implementing stringent aseptic techniques. This includes maintaining sterile fields, using proper surgical attire, and adhering to rigorous hand hygiene protocols. Furthermore, teams work together to ensure meticulous surgical site preparation, reducing the microbial load at incision sites and minimizing the risk of contamination [90]. Comprehensive preoperative patient optimization programs are becoming vital for SSI prevention. Multidisciplinary teams assess patients before surgery, addressing modifiable risk factors that can contribute to SSIs. These factors may encompass obesity, diabetes, smoking, and other comorbidities. By optimizing patients' health and addressing these risk factors, healthcare providers can significantly reduce the chances of postoperative infections [91].

Advances in surgical techniques are continually reshaping the landscape of SSI prevention. Minimally invasive and robotic-assisted surgery techniques have gained recognition for their potential to minimize tissue trauma and reduce infection risk. These approaches often result in smaller incisions, decreased blood loss, and shorter hospital stays, all contributing to a reduced risk of SSIs [92]. Integrating data analytics and surveillance into SSI prevention efforts offers a data-driven approach to identifying high-risk patients and implementing targeted interventions. Multidisciplinary teams collaborate to harness the power of big data, enabling the identification of patterns, trends, and risk factors associated with SSIs. Predictive analytics can help identify high-risk patients, allowing for pre-emptive measures and tailored prophylactic interventions [93]. Empowering patients with knowledge about their role in infection prevention encourages active participation in their care, fostering a sense of responsibility and cooperation within the healthcare team [94].

## Conclusions

In conclusion, the use of antibiotic prophylaxis in surgery represents a critical component of SSI prevention. This practice has evolved over the years, with current insights emphasizing the importance of selecting the right antibiotics, optimizing timing, and adhering to guidelines to minimize the risk of bacterial resistance. As we move forward, future directions in this field should focus on individualized approaches, incorporating advancements in diagnostics, and embracing innovative strategies such as immunomodulation to enhance SSI prevention. In an era of increasing antibiotic resistance, maintaining a delicate balance between effective prophylaxis and judicious antibiotic use is paramount. Surgeons, infection control specialists, and researchers should collaborate to refine existing protocols and explore novel methods to safeguard patient safety during surgical procedures. By doing so, we can look forward to a future where SSIs become a rarity rather than a risk, ultimately improving the overall quality of surgical care.
